# Tetanus

**Published:** 1894-03-31

**Authors:** 


### TETANUS.

Buchner[1] showed that tetanus toxin and antitoxin, when mixed and allowed to stand, do not chemically decompose each other, but act separately on an animal if injected. The toxin[2] is unaffected by serum, egg albumen, bile, saliva, or numerous microbes. It is not absorbed by the skin, nor poisonous if eaten, but is destroyed by the gastric juice, or by solar rays. Moritz[3] reports a successful case treated by Behring's serum injection. The first successful case in England is reported from Leicester[4], where 15 grammes of dried serum were dissolved in water and injected in four doses. From the second day the boy (aged 14) began to mend. Trismus neonatorum appears to be true tetanus, and has been treated by Escherish[5] with the antitoxin with some success. The duration of immunity afforded by serum injections[6] varies with the species of animal from which it was obtained. The dose required to cure an infected animal is 1,000 to 2,000 times greater than that required to produce immunity. Behring[7] has recently remarked that, as convalescents from cholera and diphtheria often carry about living bacilli harmless to themselves, so animals, treated by repeated increasing doses of tetanus toxin, develop a permanent protective. Those treated by serum containing antitoxin have only a temporary one, the tissues having gained no permanent power of reaction. Rummo, by increasing doses of strychnine, has produced in guinea pigs an immunity to tetanus.

### Cholera.

Guinea pigs can be rendered immune to intra-peritoneal injections of the cholera vibrio[8] not only by attenuated ones of the same kind, but also by those of other bacilli. Nijland[9] showed that while the bacilli soon died out in pure water, they survived in polluted, and that medicated soaps in baths in ordinary quantities had no effect, except sublimate, which, on the other hand, is useless for disinfecting evacuations.

Klein[10] finds that the flagella of the bacillus from stools, but not those from cultures, can be stained in the following way: Place a flocculus in a mixture of equal parts of absolute alcohol and anilin-water-gentian violet. Wash thoroughly in distilled water, press between two cover glasses, dry each, and mount in balsam.

The resistance of the bacilli to cold was tested by Reuk and Uffelman.[11] They only succumb after several days' freezing, and perish more rapidly if very low temperatures are employed. With regard to their dissemination in air currents,[12] it is found that when adherent to particles of dust they neither survive if floating about for any length of time nor over any considerable distance. The *British Medical Journal* points out that Stricker's[13] experiments by eating the cholera vibrio really showed its specific nature. Of six persons four showed mild symptoms, such as are frequent in epidemics.

Sanarelli[14] insisted that we cannot distinguish between cholera and other vibrios. Out of 32 varieties only four had pathogenic properties, but probably others may acquire them, hence the danger of contaminated water. He thinks there are several varieties of vibrios capable of producing cholera.

An Indian Government sanitary report by Dr. Cunningham[15] also denies that there is only one form of cholera bacillus. Several types obtained from cholera patients have been cultivated for four years and remain distinct. Besides this cases are found without bacilli, and bacilli in persons who have no cholera. He would not regard them as a cause but a consequence of the disease, and as influencing its course. Their removal may lessen the mortality.

Dehio[16] advocates the intravenous injection of salt solution as preferable to subcutaneous. It succeeds only where the toxins are not too intense, or the heart failure too great.

The method of diagnosis employed by Koch[17] is (1) a shred of mucus is placed on a cover glass, dried, and stained by carbolic fuchsin. The bacilli are seen in groups of parallel rows. (2) Shreds are placed in sterilised alkaline (one per cent. peptone and a half per cent. salt) solution for six hours at Fah. 98. The surface may be then examined as in (1). (3) Cultivations from (2) are made on agar plates, and colonies appear at Fah. 98 in ten hours. Even more definite colonies are found on gelatine, but the growth is slower. (4) The cholera red reaction is obtained in peptone cultures if pure sulphuric acid and pure cultures in peptonised water are used. (5) Intra-peritoneal injection of a twenty hours' cultivation on agar into a guinea pig. Details are given in the paper referred to and various objections met. A most important criticism has, however, been made by Sheridan Delépine[18] on the bacteriological diagnosis. He agreed that the clinical and anatomical evidence of cholera was uncertain, and showed that all Koch's tests had been answered by other organisms. Moreover, the bacilli have been found in perfectly healthy persons, and absent in cholera patients, and they vary in cilia and other ways. He concludes (1) that the bacterial evidence is not infallible; (2) water contaminated with normal dejecta causes outbreaks similar to Indian cholera; (3) the localistic views of Cunningham and Pettenkoffer have much to be said for them.

An investigation into the relations between English and Asiatic cholera is now going on because of the uncertainty of the bacterial evidence[19]. During the last summer the official reports were worded as being "positive," "negative," or "indistinguishable from true cholera." The latter phrase is declared to mean that the bacillus was found, but that the clinical evidence or the history of the patient pointed to another conclusion. Here the same precautions as to disinfection were ordered as in undoubted cases. Moreover, it is pointed out that the bacillus is occasionally not present in undoubted cholera.

On the other hand, Renvers,[20] of the Moabit Hospital, confirms the views of the German School from 122 recent cases declaring that by the bacillus alone can cholera be distinguished, and that the examination of the stools of many individuals at the time of the epidemic showed its absence in other than cholera patients.

Of the sixteen cases of cholera reported in London[21] in 1893, in only four could the bacillus be certainly demonstrated. Possibly some of the rest were true cholera, but in late stages. In Teneriffe[22] an epidemic of over one thousand cases, with a mortality of 26 per cent., ended in December, and was dealt with actively; another was stamped out at Tripoli, while a third appeared at Namur in January. The evidence connecting the Paris[23] epidemic with the use of the foul Seine water, seems to be increasing and generally accepted.

The Greenwich diarrhœa epidemic of 244 cases and 11 deaths[24] showed neither the comma bacillus nor rice water stools, and was confined to aged persons only. Some polluted well water was suspected, but evidence showed that the disease was introduced from outside, and that a new "proteus" bacillus was found in the stools of the patients, many of whom had not drunk from the well. A similar outbreak occurred at New Cross Road in January. A report on port cholera expenses to the Parliamentary Bills Committee of the British Medical Association[25], recommends that these should be paid from a general county fund, and Dr. McVail[26] points strongly to the need of legislation in Scotland to form combined sanitary port authorities in that country.

The question of the constant presence of cholera in India[27] is of international importance, and E. Hart has called attention to the part played by the Indian fairs in spreading infection, and the success attending sanitary precautions at them.

An International Conference is now sitting at Paris[28] on the defence of Europe from cholera, especially with regard to its Asiatic source and the dangers of the Mecca pilgrimage, where 500 persons died in one day last year from the water of the sacred well. The extinction of cholera might be obtained, the French delegate thinks, by ensuring pure drinking water everywhere, and meantime by regulating the Mecca pilgrimage and the Indian fairs. The sanitary organisation of India must be also considered.

### Yellow Fever.[29]

Though no special microbe can yet be detected, extensive hæmorrhages of the mucous membranes and degenerative charges in the heart, liver, and kidneys are very marked. Salicrup[30] orders 10 or 15 grains of calomel at the beginning, followed by saline and diaphoretics and general remedies. Lasting immunity is given by an attack.

### Leprosy.[31]

The question of communicability is ably reviewed by Beaven Rake. We can only give the conclusions of his most interesting paper. (1) Leprosy is probably due to a bacillus, and we must admit the possibility of its inoculation; (2) experimental inoculations have never succeeded beyond the possibility of doubt; (3) it has not been proved that vaccination has conveyed leprosy; (4) the evidence tends to show that communication from one person to another must be rare and under exceptional conditions; (5) leprosy has steadily decreased in many places without segregation and *vice versa*; (6) Immigation of lepers into leper-free countries has not in recent times been followed by any spread of the disease; (7) practically it is less infectious than tuberculosis and requires no greater precautions.

Nerve-stretching[32] has been performed with great success for the relief of the symptoms in more than 250 cases at the suggestion of Dr. K. McLeod, considerable restoration of power being obtained. Treatment by the oil of hydrocarpus inebrians by Dr. Bhau Daji[33] is said to have yielded remarkable results, and is now being tested more fully.

### Anthrax.

Galtier[34] has investigated the causes which increase the virulent action of anthrax. Previous disease, intravenous injections of water, the presence of attenuated anthrax, streptococci, or other microbes may render animals susceptible which were otherwise immune, and reciprocally the anthrax invasion may increase the power of otherwise harmless parasites, The spread of anthrax, cholera, typhoid, phthisis, ophthalmia, diphtheria, syphilis, and leprosy by flies is pointed out as a frequent possibility by Sir W. Moore.[35].

### Beri-beri.

Works on this subject have been accumulating of late, those of Dr. Bentley and Pekelharing[36] are perhaps the most important. The bacteriology is quite uncertain. It primarily affects the nervous system, causing paralysis and anæsthesia. Sore throat occurs at some stage of the disease, but neither abnormal temperatures nor nephritis are present.

[1] Berlin Klin. Woch., No. 4, 1894. [2] B. M. J., Feb. 3, 1893. [3] Am. J. Med. Sc., Nov., 1893. [4] Lancet, Jan. 27, 1894. [5] Med. Rec., Jan. 27, 1894. [6] Berlin Klin. Woch., 49, 52, 1893. [7] Deuts. Med. Woch., No. 48, 1893, and Rif. Med., Jan. 10. [8] B. M. J., Jan. 20, 1894. [9] Am. J. Med. Sc., Dec., 1893. [10] Centrall. f. Bakt., XIV. B. 19. [11] Am. J. Med. Sc., Jan., 1894. [12] Am. J. Med. Sc., Feb., 1894. [13] B. M. J., Jan. 13, 1894. [14] B. M. J., Dec. 9, 1893. [15] Lancet, Feb. 10, 1894. [16] B. M. J., Jan. 13, 1894. [17] Zeitsch. f. Hyg., XIV., 2, 1893.; Practic., Dec., 1893. [18] B. M. J., Jan. 20, 1894. [19] Practit., Dec., 1893. [20] Lancet, Jan. 27, 1894. [21] Lancet, Feb. 17. 1894. [22] Lancet, Feb. 27, 1894. [23] Lancet, Feb. 3, 1894. [24] B. M. J., Dec., 1893, and Feb. 10, 1893. [25] B. M. J., Jan., 1894. [26] Glas. Med. J., Jan., 1894. [27] B. M. J., Feb. 17, 1894. [28] Lancet, Feb. 10; B. M. J., Jan. 27, Feb. 3, 1894. [29] Med. Rec., Jan. 27, 1894. [30] Ther. Gaz., Dec., 1893. [31] Med. Rec., Dec. 2, 1893. [32] Lancet, Feb. 17. [33] B. M. J., Dec. 30. [34] Med. Week, Jan. 5, 1894. [35] Am. J. Med. Sc., Jan., 1894. [36] Lancet, Jan. 20; B. M. J., Feb. 10, 1894.

